# Management of paraesophageal hiatus hernia: recommendations following a European expert Delphi consensus

**DOI:** 10.1007/s00464-023-09933-8

**Published:** 2023-02-27

**Authors:** Stephan Gerdes, Sebastian F. Schoppmann, Luigi Bonavina, Nicholas Boyle, Beat P. Müller-Stich, Christian A. Gutschow, the Hiatus Hernia Delphi Collaborative Group, Suzanne Sarah Gisbertz, Ferdinand Köckerling, Thorsten G. Lehmann, Dietmar Lorenz, Frank Alexander Granderath, Riccardo Rosati, Christoph Wullstein, Lars Lundell, Edward Cheong, Philippe Nafteux, Stefano Olmi, Stefan Mönig, Matthias Biebl, Jessica Leers, Joerg Zehetner, Ivan Kristo, Richard George Berrisford, Ognjan M. Skrobić, Aleksandar P. Simić, Manuel Pera, Peter Philipp Grimminger, Ines Gockel, Konstantinos Zarras, Vincent Bernard Nieuwenhuijs, James A. Gossage, Mark i. van Berge Henegouwen, Hubert J. Stein, Sheraz R. Markar, Willem Eduard Hueting, Eduardo M. Targarona, Jan Johansson, Graeme D. Macaulay, Bas P.L. Wijnhoven, Frank Benedix, Stephen E. Attwood, Arnulf Heinrich Hölscher, Pablo Priego, Karl-Hermann Fuchs, Misha D.P. Luyer, Ewen A. Griffiths, Torgeir Thorson Søvik, Dimitrios Theodorou, Bruno Sgromo, Jarmo A. Salo, Rishi Singhal, Anders Thorell, Giovanni Zaninotto, Marko Bitenc, Xavier Benoit D’journo, Grant M. Fullarton, Thomas Horbach

**Affiliations:** 1 Department of Surgery and Transplantation, University Hospital Zurich, Rämistrasse 100, 8091 Zurich, Switzerland; 2 Department of Surgery, Medical University of Vienna, Vienna, Austria; 3 Division of General and Foregut Surgery, IRCCS Policlinico San Donato, University of Milan, Milan, Italy; 4 RefluxUK, London, UK; 5 Department of General, Visceral and Transplant Surgery, University Hospital, Heidelberg, Germany; 6 Department of Surgery, Amsterdam UMC Location University of Amsterdam, Amsterdam, The Netherlands; 7 Vivantes Humboldt-Hospital, Academic Teaching Hospital of Charité University Medicine, Berlin, Germany; 8 Allgemein- und Viszeralchirurgie Friedrichshafen & Tettnang, Friedrichshafen, Germany; 9 Allgemein-, Viszeral- und Thoraxchirurgie, Klinikum Darmstadt, Darmstadt, Germany; 10 Department of General and Visceral Surgery, Hospital Neuwerk, Mönchengladbach, Germany; 11 San Raffaele Hospital and San Raffaele Vita-Salute University, Via Olgettina, 60, 20132 Milan, Italy; 12 Department of Visceral and Minimalinvasive Surgery, Helios Hospital Krefeld, Krefeld, Germany; 13 Division of Surgery, CLINTEC, Karolinska Institutet, Stockholm, Sweden; 14 UGI and General Surgery Department, Norfolk and Norwich University Hospital, Colney lane, Norwich, UK; 15 Department of Thoracic Surgery, University Hospitals Leuven, Louvain, Belgium; 16 Department of General and Oncologic Surgery, Center for Obesity Surgery, Policlinico San Marco, Zingonia, BG Italy; 17 Geneva University Hospitals, Geneva, Switzerland; 18 Department of Surgery, Ordensklinikum Linz, Linz, Austria; 19 Klinik für Allgemein-, Viszeral-, Tumor- und Transplantationschirurgie, Universitätsklinikum Köln, Köln, Germany; 20 Hirslanden Klinik Beau-Site Bern, Bern, Switzerland; 21 Department of General Surgery, Medical University of Vienna, Wien, Italy; 22 University Hospitals Plymouth, Devon, UK; 23 University Hospital for Digestive Surgery, Clinical Center of Serbia, School of Medicine, University of Belgrade, Belgrade, Serbia; 24 Section of Gastrointestinal Surgery, Department of Surgery, Hospital del Mar, Hospital del Mar Medical Research Institute (IMIM), Universitat Autònoma de Barcelona, Barcelona, Spain; 25 Klinik für Allgemein-, Viszeral- und Transplantationschirurgie, Universitätsmedizin Mainz, Mainz, Germany; 26 Department of Visceral, Transplant, Thoracic and Vascular Surgery, University Hospital of Leipzig, Leipzig, Germany; 27 Klinik für Viszeral-, Minimalinvasive und Onkologische Chirurgie, Marien Hospital Düsseldorf, Düsseldorf, Germany; 28 Department of Surgery, Isala, Zwolle, The Netherlands; 29 St Thomas’ Hospital, London, UK; 30 Department of Surgery, Amsterdam UMC, University of Amsterdam, Amsterdam, The Netherlands; 31 Paracelsus Private Medical School, Nuremberg, Germany; 32 Nuffield Department of Surgery, University of Oxford, Oxford, UK; 33 Alrijne Medical Center Leiden, Leiden, The Netherlands; 34 Gastroinestianl Surgical Unit Hospital Santpau, Barcelona, Spain; 35 Surgery & Gastroenterology, Department of Clinical Sciences Lund, Lund University, Skane University Hospital, Lund, Sweden; 36 Regional Oesophago-gastric Unit Belfast City Hospital, Belfast, Northern Ireland; 37 Department of Surgery, Erasmus University Medical Center, Rotterdam, The Netherlands; 38 Department of Upper GI Surgery, University Hospital Magdeburg, Magdeburg, Germany; 39 Durham Universitiy, Durham, UK; 40 Elisabeth Hospital Essen, Elisabethen Hospital, Frankfurt, Germany; 41 Division of Esophagogastric and Bariatric Surgery, Ramón y Cajal University Hospital, Madrid, Spain; 42 Laboratory for Interventioneal and Experimental Endoscopy, University of Wuerzburg, Würzburg, Germany; 43 Department of Surgery, Catharina Hospital, Eindhoven, The Netherlands; 44 Department of Upper GI Surgery, Queen Elizabeth Hospital Birmingham, Mindelsohn Way, Edgbaston, UK; 45 Department of Pediatric and Gastrointestinal Surgery, Oslo University Hospital Ullevål, Oslo, Norway; 46 Upper GI Surgery Unit, University of Athens School of Medicine, Athens, Greece; 47 Oxford University Hospitals, Oxford, UK; 48 Department of General Thoracic and Esophageal Surgery, Helsinki University Central Hospital, Helsinki, Finland; 49 Birmingham Heartlands Hospital, University Hospital Birmingham, Birmingham, UK; 50 Department of Surgery, Ersta Hospital, Karolinska Institutet, Stockholm, Sweden; 51 Department of Surgery and Cancer, Imperial College, London, UK; 52 Kirurgija Bitenc d.o.o, Vilharjev Podhod 1, 1000 Ljubljana, Slovenia; 53 Thoracic Surgery Department, Aix-Marseille University, Marseille, France; 54 Department of Upper GI Surgery, Glasgow Royal Infirmary, Glasgow, Scotland; 55 Viszera Chirurgie-Zentrum München, Munich, Germany; 56 Department of Surgery, Odense University Hospital University of Southern Denmark, Odense, Denmark; 57 Cancer Center Amsterdam, Amsterdam, The Netherlands

**Keywords:** Hiatus hernia, Paraesophageal hernia, Surgical technique, Mesh, Fundoplication, Delphi survey

## Abstract

**Aims:**

There is considerable controversy regarding optimal management of patients with paraesophageal hiatus hernia (pHH). This survey aims at identifying recommended strategies for work-up, surgical therapy, and postoperative follow-up using Delphi methodology.

**Methods:**

We conducted a 2-round, 33-question, web-based Delphi survey on perioperative management (preoperative work-up, surgical procedure and follow-up) of non-revisional, elective pHH among European surgeons with expertise in upper-GI. Responses were graded on a 5-point Likert scale and analyzed using descriptive statistics. Items from the questionnaire were defined as “recommended” or “discouraged” if positive or negative concordance among participants was > 75%. Items with lower concordance levels were labelled “acceptable” (neither recommended nor discouraged).

**Results:**

Seventy-two surgeons with a median (IQR) experience of 23 (14–30) years from 17 European countries participated (response rate 60%). The annual median (IQR) individual and institutional caseload was 25 (15–36) and 40 (28–60) pHH-surgeries, respectively. After Delphi round 2, “recommended” strategies were defined for preoperative work-up (endoscopy), indication for surgery (typical symptoms and/or chronic anemia), surgical dissection (hernia sac dissection and resection, preservation of the vagal nerves, crural fascia and pleura, resection of retrocardial lipoma) and reconstruction (posterior crurorrhaphy with single stitches, lower esophageal sphincter augmentation (Nissen or Toupet), and postoperative follow-up (contrast radiography). In addition, we identified “discouraged” strategies for preoperative work-up (endosonography), and surgical reconstruction (crurorrhaphy with running sutures, tension-free hiatus repair with mesh only). In contrast, many items from the questionnaire including most details of mesh augmentation (indication, material, shape, placement, and fixation technique) were “acceptable”.

**Conclusions:**

This multinational European Delphi survey represents the first expert-led process to identify recommended strategies for the management of pHH. Our work may be useful in clinical practice to guide the diagnostic process, increase procedural consistency and standardization, and to foster collaborative research.

**Supplementary Information:**

The online version contains supplementary material available at 10.1007/s00464-023-09933-8.

Optimal treatment of paraesophageal hiatus hernia (pHH) remains a highly debated topic in upper-gastrointestinal (UGI) surgery. Numerous aspects regarding both the diagnostic work-up and the surgical management of this clinical entity are not broadly accepted, and even experts disagree on critical components including the application of surgical meshes for hiatal reinforcement, the indication for complementary sphincter augmentation and the diagnosis and treatment of short esophagus.

Based on our own clinical experience, we hypothesized that the current surgical practice may reflect those uncertainties. Since there are no uniform recommendations from national or international societies on this topic, we found it pertinent to perform a Delphi survey among recognized experts in UGI-surgery to identify recommended strategies.

## Material and methods

### Expert panel

Inclusion criteria for invited experts were ≥ 10 years of experience in UGI-surgery, an annual institutional caseload of ≥ 30 hiatal hernias, and a specialty interest in UGI-surgery as evidenced by recent (within the last 10 years) publications in the field. From their personal professional network, the lead authors of this project worked out a list of potential participants fulfilling the above criteria. This list was supplemented by board members of the European Foregut Society (EFS), a recently founded scientific society with a specific focus on benign esophago-gastric disease.

### Delphi survey

To minimize bias, the focus was strictly on elective (planned) repair of non-revisional pHH; other hernia types, emergencies and recurrences were considered outside the scope of this work. PHH was defined as Skinner type II (true paraesophageal), type III (mixed sliding/axial and paraesophageal), and type IV (paraesophageal and herniation of other abdominal organs). The lead authors designed a 33-question survey to elicit respondent feedback on the following parameters: personal and institutional experience, diagnostic work-up, indications, technical details of hiatal repair (access routes, surgical dissection and reconstruction), and postoperative follow-up (Online Appendix 1). An online survey tool (SurveyMonkey, Palo Alto, CA, USA) was employed to disseminate the survey and to collect answers. In May 2021, experts were invited to participate via an email containing the study protocol, the expected number of Delphi rounds, and the anticipated time commitment. After agreeing to participate, experts were provided with access to each Delphi round via secure, institute-to-institute email. The attendees were also invited to leave comments on each question and to suggest changes to the wording. Throughout the Delphi survey, voting and commenting was conducted anonymously.

In both Delphi rounds, participants were asked to rank their agreement on each question using a 5-point Likert scale. Two scale variations were employed, the first indicated level of recommendation (1 = strongly recommended, 2 = recommended, 3 = neither recommended nor discouraged, 4 = discouraged, 5 = strongly discouraged) whilst the second informed the consent with which technical steps are performed by the participant (1 = a great deal, 2 = considerably, 3 = moderately, 4 = slightly, 5 = not at all).

After completion of Delphi round 1, the lead authors adapted the results according to the expert’s suggestions to create the next questionnaire. In Delphi round 2, the percentage of concordance (“strongly recommended” or “recommended” and “a great deal” or “considerably”) from the preceding round were visible, enabling experts to re-vote in consideration of previous results (Online Appendix 2).

Experts were given two weeks to complete each round. Two reminders were sent, the first one week after opening and the second two days before closing of each round. Data collection took place from May 2021 to September 2021.

### Data analysis

Items of the questionnaire were defined as “recommended” if positive concordance among participants was > 75% (“strongly recommended” or “recommended” and “a great deal” or “considerably”) on a given question. Likewise, items were defined as “discouraged” if negative concordance among participants was > 75% (“discouraged” or “strongly discouraged” and “slightly” or “not at all”). Items with lower positive or negative concordance levels were categorized as “acceptable” (neither recommended nor discouraged). Data were analyzed using descriptive statistics and expressed as percentage of agreement and median (IQR) using SPSS version 26.0 (IBM Inc., Chicago, Ill, USA). No IRB approval or written consent was required for the paper.

## Results

### Participants

One hundred twenty-one European experts for upper-GI surgery were invited for round 1 and 2 of the Delphi, and 72 surgeons across 17 countries responded (response rate 60%). Details of participants are displayed in Table 1.

**Table 1:** Details of the participants

	(%)	*n*
Institution		
University Hospital	67	48
Maximum Care Hospital	8	6
Teaching Hospital	14	10
General Hospital	1	1
Private Hospital	10	7
Position		
Head of department	39	28
Senior consultant	40	29
Consultant	11	8
Attending surgeon	3	2
Retired	7	5
Institutional caseload per year		
Mean		52
Median (IQR)		40 (28–60)
Individual caseload per year		
Mean		23
Median (IQR)		25 (15–36)

### Definition of paraesophageal hiatus hernia

There was agreement (91%) that pHH should be defined as the presence of a hernia sac extending from the abdominal cavity and/or bursa omentalis through the hiatus into the paraesophageal mediastinum and containing a variable portion of stomach. In contrast, the sole presence of a hernia sac or of gastric prolapse into the mediastinum was not considered as a suitable definition of pHH.

### Preoperative diagnostic work-up and indication for surgery

**Fig. 1:**
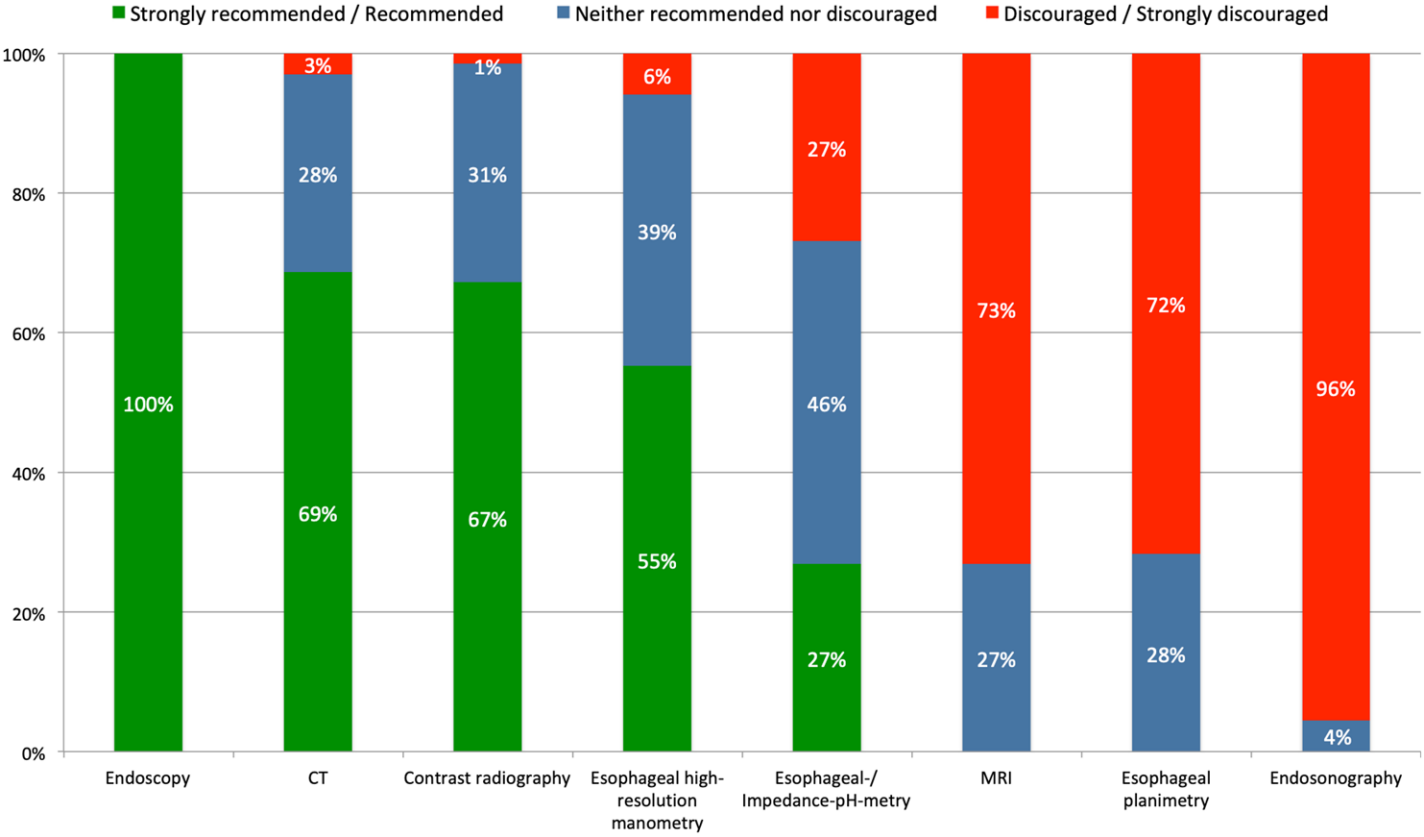
Expert recommendations for diagnostic work-up for pHH

Upper-GI endoscopy was formally “recommended” and esophageal endosonography was “discouraged” as preoperative diagnostic tests. In contrast, most other diagnostic tools (CT scan, contrast radiography, esophageal manometry, (impedance-) pH-testing, MRI, and esophageal planimetry) were categorized as “acceptable” (Fig. 1).

Surgery was the “recommended” therapeutic strategy in symptomatic and anemic patients independent of biological age (> or < 70 years). Conversely, indication for surgery was “acceptable” in patients with no or minor symptoms.

### Access routes and steps of surgical dissection

Laparoscopy was the preferred access route of most (89%) participants. Other surgical access techniques (robotic-assisted laparoscopy (8%), thoracoscopy (1%), thoracotomy (1%)) were rarely used.

“Recommended” steps of the surgical dissection phase entailed resection of the hernia sac, visualization of both vagal nerves, resection of the retrocardiac lipoma, preservation of the crural fascia and of the pleural sac; all other details of surgical dissection were categorized as “acceptable” (Table 2).

**Table 2:** Expert recommendations of surgical dissection for pHH repair

	Strongly recommended/Recommended (%)	Neither recommended nor discouraged (%)	Discouraged/Strongly discouraged (%)	Overall assessment
Dissection of hernia sac from mediastinum	100	0	0	Recommended
Visualization of both vagal nerves	96	4	0	Recommended
Preservation of the crural fascia	96	1	3	Recommended
Resection/mobilization of retro-cardial lipoma	93	4	3	Recommended
Resection of hernia sac	90	7	3	Recommended
Preservation of pleural sac	84	10	6	Recommended
Repositioning of hernia sac contents during dissection of hernia sac from the mediastinum	73	24	3	Acceptable
Mobilization of gastric fundus	67	21	12	Acceptable
Preservation of aberrant left hepatic artery	67	27	6	Acceptable
Resection/mobilization of pre-cardial fat-pad	66	21	13	Acceptable
Repositioning of hernia sac contents as initial step of procedure	63	24	13	Acceptable
Intraoperative positioning of a large-bore esophageal tube	37	37	25	Acceptable
Preservation of hepatic branches of vagus nerves	28	52	19	Acceptable
Intraoperative endoscopy	25	58	16	Acceptable
Preservation of pulmonary branches of vagus nerves	21	58	21	Acceptable
Visualization of pulmonary veins	6	48	46	Acceptable

### Surgical reconstruction

“Recommended” steps of surgical reconstruction included suture repair of the hiatus and lower esophageal sphincter augmentation. All other steps of surgical reconstruction were categorized as “acceptable” (Table 3).

**Table 3:** Expert recommendations for technical steps during reconstruction in pHH repair

	Strongly recommended/Recommended (%)	Neither recommended nor discouraged (%)	Discouraged/Strongly discouraged (%)	Overall assessment
Suture repair	100	0	0	Recommended
Antireflux procedure	96	4	0	Recommended
In case of short esophagus: Esophageal lengthening procedure (Collis or other)	51	36	13	Acceptable
Positioning of large-bore esophageal tube	45	37	18	Acceptable
Gastropexy	40	33	27	Acceptable
Use of mesh	25	52	22	Acceptable
Postoperative wound drain	24	25	51	Acceptable
Postoperative gastric decompression tube	16	25	58	Acceptable
Postoperative chest drain	6	25	69	Acceptable
Ligamentum teres to reinforce hiatal repair	4	43	52	Acceptable
Use of relaxing diaphragmatic incisions	4	39	57	Acceptable
Left hepatic lobe (hepatic shoulder) to reinforce hiatal repair	3	30	67	Acceptable

### Hiatoplasty (crurorrhaphy and mesh augmentation)

**Fig. 2:**
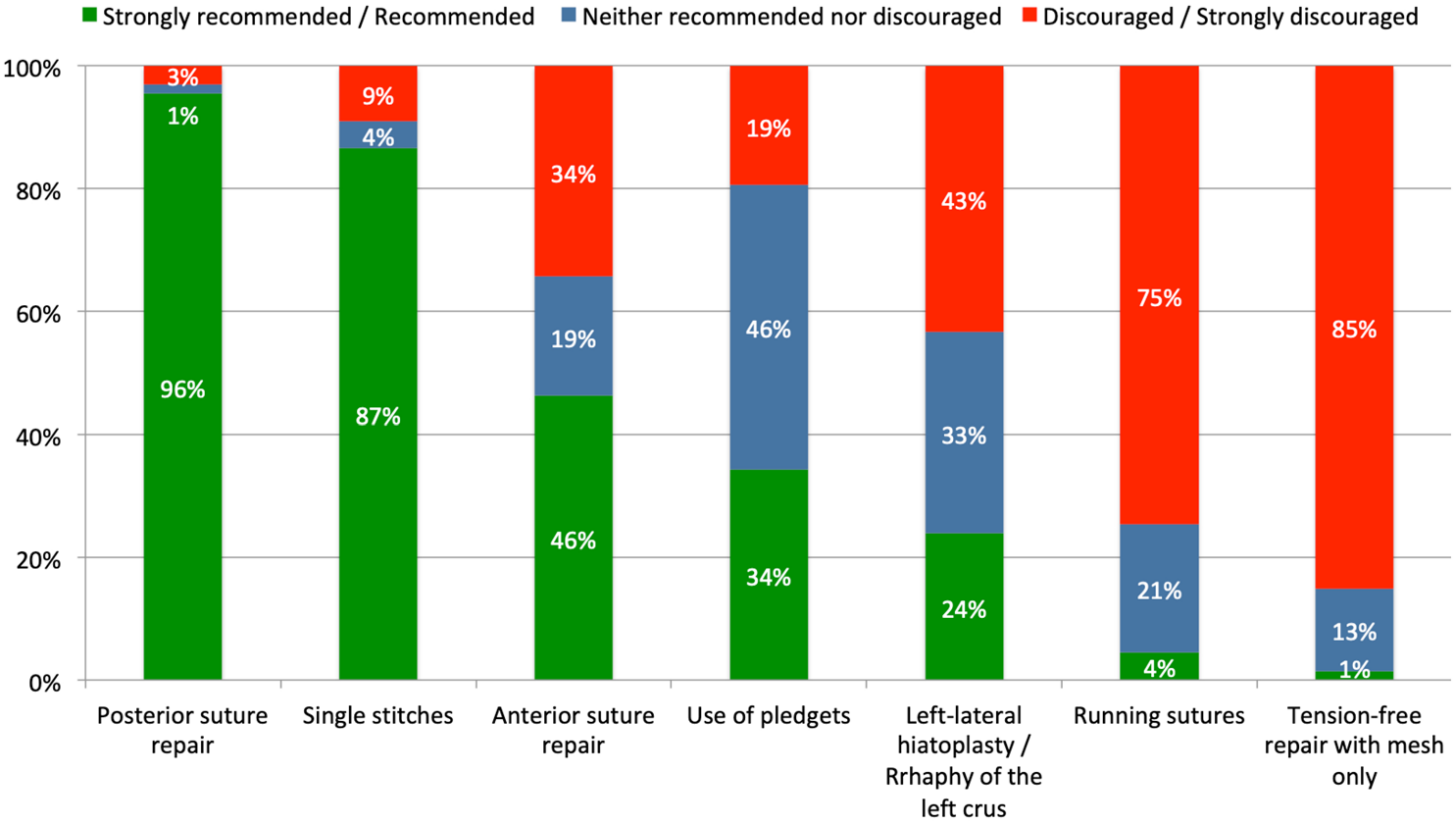
Expert recommendations for techniques for hiatoplasty in pHH repair

“Recommended” techniques for hiatoplasty were posterior crurorraphy, use of single stitches and non-resorbable braided suture material (size 0 or 2-0). In contrast, crurorrhaphy with running sutures and diaphragmatic relaxing incisions were “discouraged”. All other technical details of hiatoplasty (anterior and left-lateral crurorraphy, use of pledgets) were classified as “acceptable” (Fig. 2).

Most participants (72%) perform selective mesh augmentation (always 10%, never 18%), and there were no “recommended” or “discouraged” strategies regarding material, placement, shape, and fixation of mesh. Among selective mesh-users, indications were fragile diaphragmatic musculature (78%), large hiatus defects (73%), and recurrent hernia (73%). Preferred materials were synthetic absorbable, synthetic non- or partially absorbable, and biological meshes in 47%, 39%, and 25%, respectively, and mesh placement is performed posteriorly open (u-shape), anteriorly open (u-shape), completely encircling the esophagus (circular), and on posterior hiatoplasty only in 54%, 36%, 26%, and 5%, respectively. In contrast, most participants (81%) agreed to use sutures for mesh fixation, while other techniques (tacks 19%, fibrin glue 31%) did not reach concordance.

Of note, a relevant percentage of surgeons encountered mesh-related complications such as erosion, stenosis, mesh migration in own or referred patients. (Table 4).

**Table 4:** Mesh-associated complications encountered by participants

Complication	In own patients (%)	In referred patients (%)	Never (%)
Mesh erosion (esophagus, stomach, or esophago-gastric junction)	19	72	19
Mesh erosion to other organs (aorta, lung)	3	27	72
Stenosis distal esophagus/esophago-gastric junction	15	71	24
Mesh migration	14	59	35
Mesh infection	10	35	61
Pericardial tamponade	6	7	89
Pleural hemorrhage	7	7	86
Perioperative hemorrhage caused by mesh fixation	6	10	85
Pneumothorax	27	13	68
Chronic pain	17	35	55
Seroma formation	31	22	56

### Lower esophageal sphincter augmentation and management of short esophagus

**Fig. 3:**
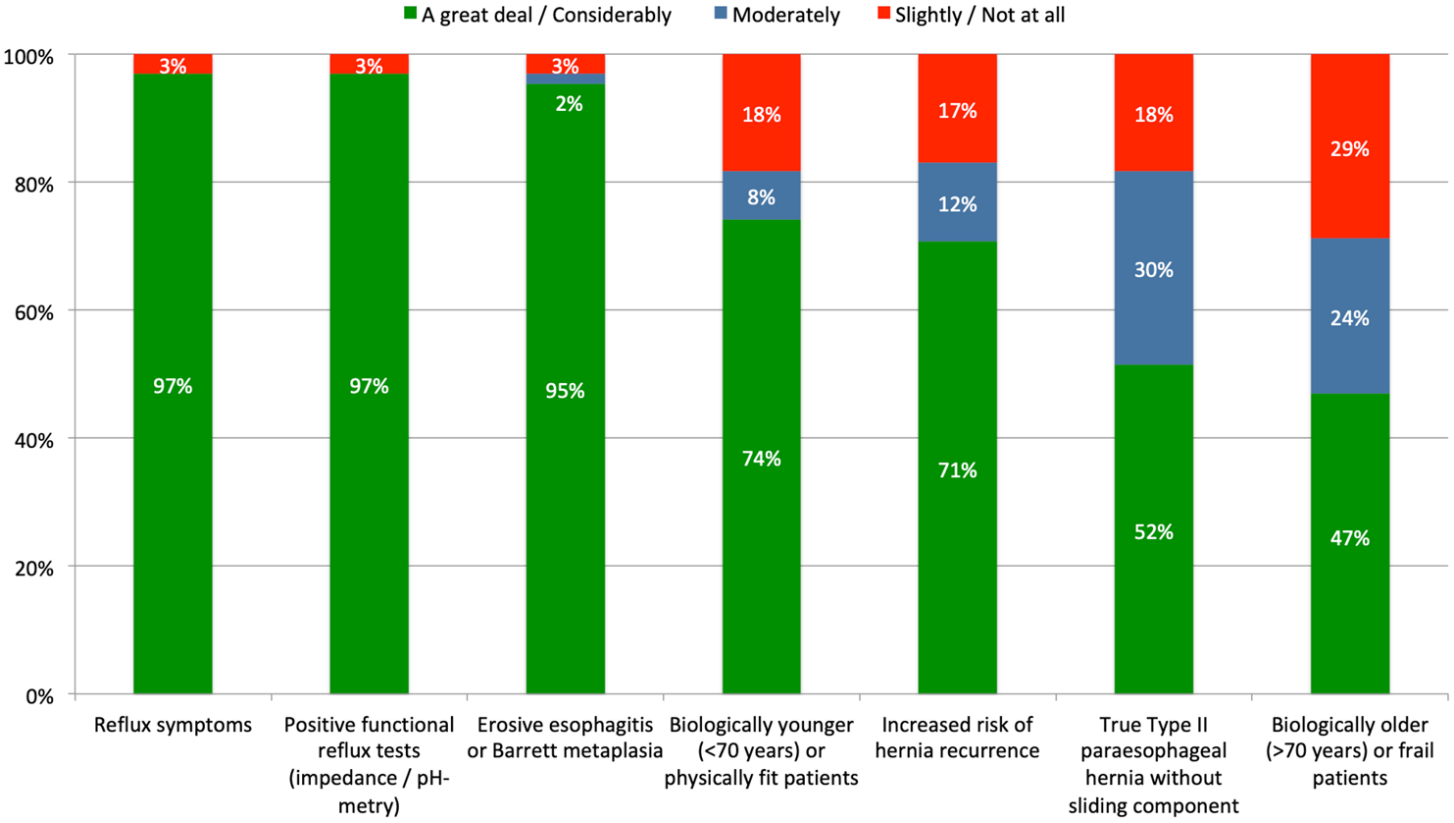
Expert agreements for indications for LES augmentation in pHH repair

**Fig. 4:**
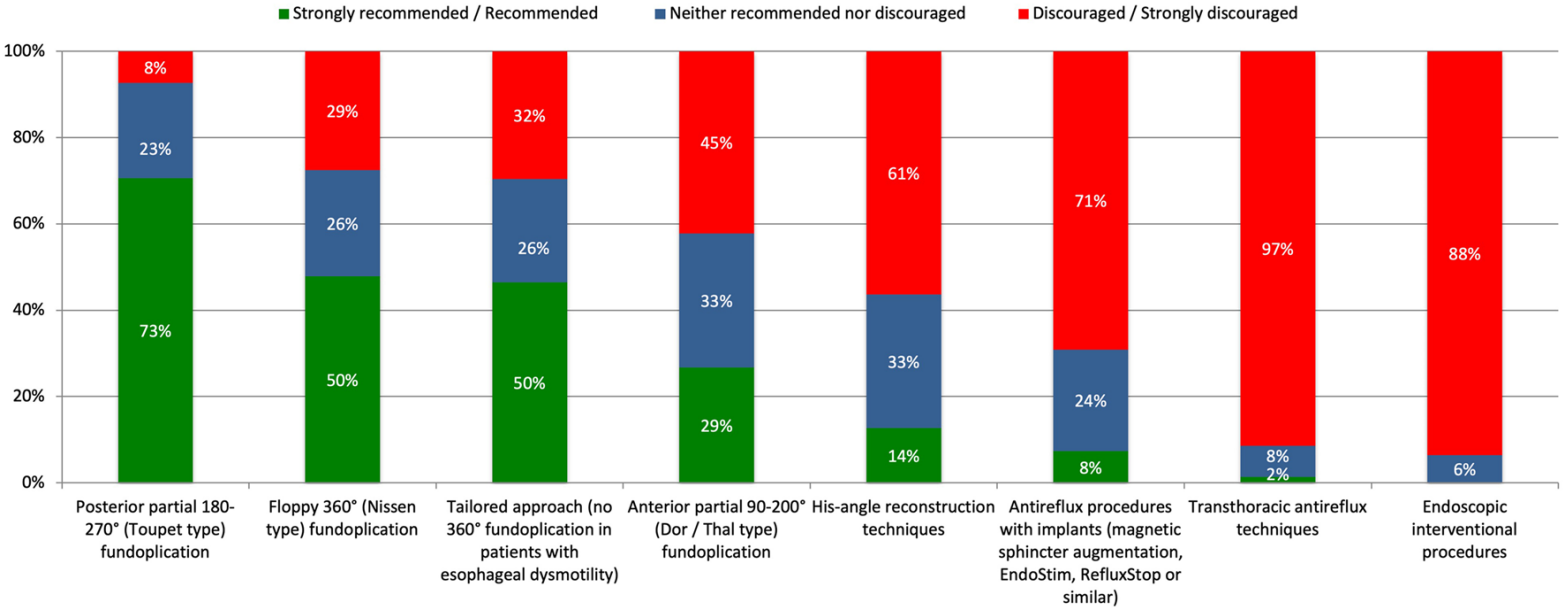
Expert recommendations for antireflux procedures in pHH repair

There was a high level of concordance (96%) among participants to perform an additional augmentation of the lower esophageal sphincter (LES) in all (64%) or selected (35%) patients. “Recommended” indications for LES augmentation were the presence of reflux symptoms (97%), erosive esophagitis or Barrett’s metaplasia (95%), and positive functional tests (97%). In contrast, biological age, increased risk for HH recurrence, and true Type II pHH did not impact the indication for LES augmentation (Fig. 3). Likewise, except “discouraged” transthoracic and interventional/endoscopic approaches, most LES augmentation techniques (total, partial and tailored fundoplication, techniques involving surgical implants) were categorized “acceptable” (Fig. 4).

Only a minority (44%) of participants agreed that short esophagus (SE) is a relevant finding during pHH repair (not sure: 28%, disagree: 28%). Collis gastroplasty and simple gastropexy were the only “acceptable” surgical techniques; all other procedures were “discouraged”.

### Follow up and clinical definition of recurrence

**Fig. 5:**
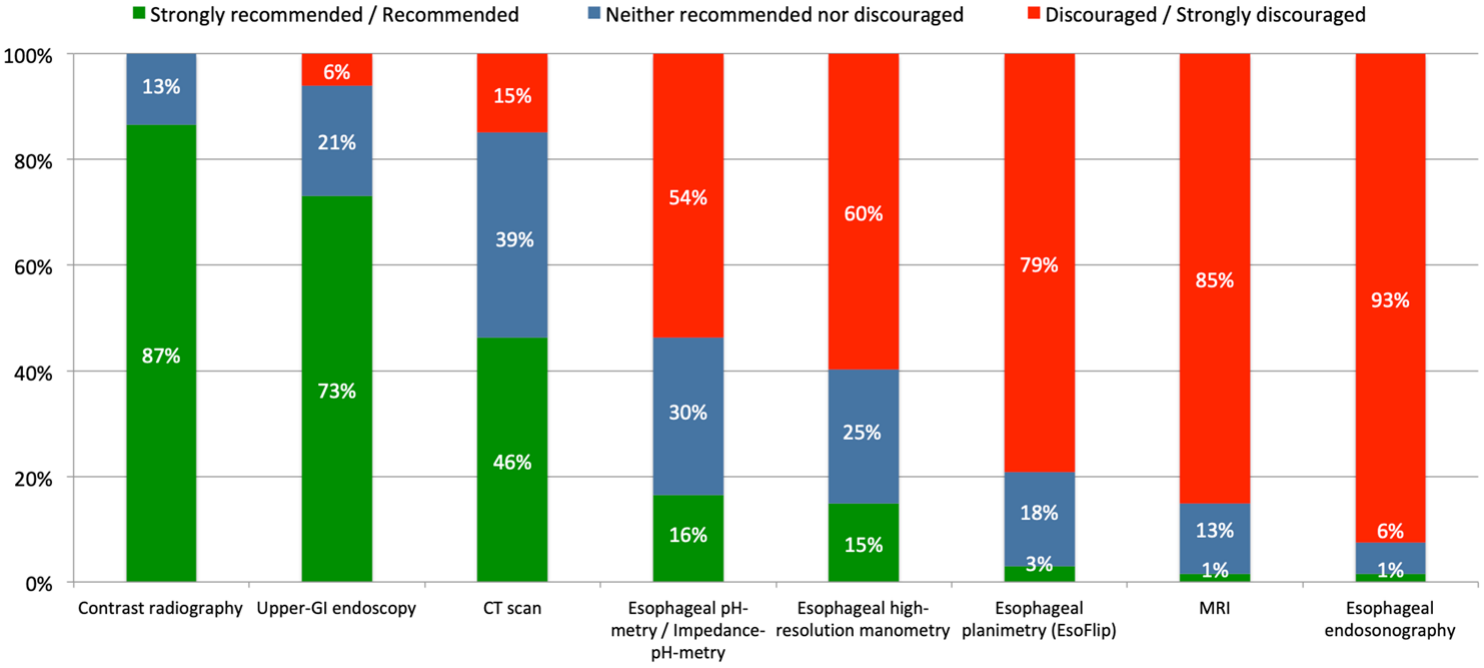
Expert recommended diagnostic procedures to exclude recurrence after pHH repair

Contrast radiography was formally “recommended” as a diagnostic tool for clinical follow-up and “neutral” recommendation level was reached for upper-GI endoscopy, CT scan, esophageal manometry, pH-metry. In contrast, MRI, esophageal planimetry and endosonography were “discouraged” (Fig. 5).

No recommendation was reached regarding the anatomical definition of recurrence and there was no negative or positive concordance > 75% to define clinical failure of pHH repair (Table 5).

**Table 5:** Anatomical und clinical definition of recurrent hernia despite expert opinion

Anatomical definition of recurrent hernia	(%)	*n*
Any evidence of gastric tissue above the diaphragm	36	24
At least 1 cm of gastric tissue above the diaphragm	9	6
At least 2 cm of gastric tissue above the diaphragm	27	18
At least 3 cm of gastric tissue above the diaphragm	19	13
At least 4 cm of gastric tissue above the diaphragm	4	3
Other	4	3

Clinical definition of recurrent hernia	(%)	*n*
Radiological and/or endoscopic evidence of gastric tissue above the diaphragm is sufficient to define recurrent hiatus hernia	39	26
Clinical evidence (symptoms) is sufficient to define recurrent hiatus hernia	0	0
Radiological and/or endoscopic (gastric tissue above the diaphragm) and clinical (symptoms) evidence is required to define recurrent hiatus hernia	58	39
Other	3	2

## Discussion

We performed this comprehensive survey among experts for UGI-surgery with the aim to identify recommended strategies for the treatment of pHH repair. As a result, we were able to identify high rates of concordance regarding indications for surgery, preoperative work-up, and several technical-surgical steps of hiatal dissection and reconstruction. However, an important lesson from this survey is that only few basic strategies for pHH are currently unanimously “recommended” or “discouraged”. Thus, huge uncertainties remain for many classical adjuncts such as the use of meshes, sphincter augmentation, and gastropexy, but also for the management of Short Esophagus and the correct definition and diagnosis of recurrence or failure. The limited concordance across elementary steps of pHH treatment observed in this European expert Delphi may reflect the influence of different surgical schools, but certainly mirrors a general therapeutic uncertainty caused by a notorious lack of reliable data and scientific evidence in the field.

The strengths of our survey include a large number of participating experts, a high response rate, and a well-defined index procedure (pHH). Furthermore, the modified Delphi approach enabled us to adapt and specify questions during the survey to achieve a sharper reflection of predominant recommendations.

The questionnaires were exclusively targeted at UGI surgeons with a high surgical caseload, the majority of whom holding appointments as chiefs or senior consultants in university or teaching hospitals. We selected pHH repair as the index procedure because this entity is highly prevalent (about 20% of all HH cases). In addition, exclusion of type I HH allowed for a more precise interpretation of results and conclusions. Our focus on pHH lies in contrast with some of the published literature, which comprises five surveys from the last decade, addressed to either members of the Society of American Gastrointestinal and Endoscopic Surgeons (SAGES) [1, 2], the European Association for Endoscopic Surgery (EAES), and members of the Swiss Society of Visceral Surgeons (SGVC) [3–5]. Of note, except for the European and the Swiss studies, which focused on type II-IV and type III HH [3] the SAGES surveys were designed to gather data on all types of HH including gastroesophageal reflux disease. Therefore, comparison with our results remains partly elusive. In addition, two retrospective population-based analyses on outcomes of mesh use in paraesophageal (type II–IV) HH repair using the American College of Surgeons National Surgical Quality Improvement Program (NSQIP) database have been recently reported [6, 7]. The prospective multi-national HERNIAMED data collection included 5462 paraesophageal hernia repairs and still remains another important source of information on the subject [8].

Conventional laparoscopy was the preferred surgical access route (92%), which compares favorably to data from the NSQIP and HERNIAMED databases [6, 8], as well as the SAGES and SGVC surveys [1, 2, 5] In accordance with the existing literature, our study confirms that transthoracic approaches for pHH have been largely abandoned. Of note, robotic-assisted laparoscopic surgery is still quite unpopular among European experts (8% preference), contrasting our recent survey among members of the SGVC (41% preference) [5].

The use of mesh to reinforce hiatal repairs remains a highly controversial subject, and a recent meta-analysis of RCT’s did not show any advantage of mesh augmentation over sutured hiatal closure [9]. Nevertheless,—as in the recent SGVC survey [5]—more than 80% of our participants use meshes in all or selected patients. Data from the HERNIAMED registry confirmed a rather constant, but much lower utilization rate of meshes in paraesophageal hernia repair in Austria, Germany, and Switzerland (33.0% and 38.9% in 2013 and 2019, respectively) [8], whereas in the US, this rate even decreased between from 45% in 2010 to 36% in 2017 [7]. It must be kept in mind that the current scientific evidence regarding meshes is extremely fragmented owing to different materials, shapes, fixation techniques, and follow-up periods, and the exact incidence of the much-feared mesh-related complications is not precisely known and estimated to 1–2% according to a large systematic review [10–17]. Nevertheless, more than 80% of our participants stated that they have encountered patients with mesh complications such as erosion. In contrast to earlier research, biological meshes play a minor role in the current surgical armamentarium, probably owing to the disappointing long-term results from two RCT’s [14, 16]. Thus, most of our participants chose synthetic non-absorbable mesh, which is in line with other recent surveys [3–5]. In this context, the significance of synthetic long-term absorbable materials remains unclear. Recent retrospective cohort studies have shown promising results, but long-term follow-up is currently not available [18–20].

Augmentation of the lower esophageal sphincter is a frequently performed adjunct to pHH repair and was formally “recommended” by our participants. Our results confirm recent data from the multi-institutional HERNIAMED registry reporting additional sphincter augmentation in paraesophageal hernia repair in 60–70% [8, 21]. However, routine and selective sphincter augmentation is performed by 64% and 35% of our participants, respectively, which contrasts the 84% (routine) and 9% (selective) sphincter augmentation rates in the EAES survey [3]. We assume that these differences reflect the rather weak scientific evidence for additional sphincter augmentation in the literature, which is mainly based on a single RCT [17], and a number of case series and small cohort studies [22].

As in a previous survey [5], there was no concordance among our experts regarding gastro- or fundo-phrenicopexy, probably due to the limited and conflicting evidence for this surgical adjunct in pHH repair [22–25]. Similarly, we found a mixed attitude towards short esophagus: 56% of participants were either unsure or disagreed that esophageal shortening represents a relevant finding during pHH repair. Of those confirming the importance of esophageal foreshortening, 67% agreed that esophageal lengthening (Collis) procedure and fundoplication around the neo-esophagus should be performed in this situation, which is in line with current expert recommendations [5, 26]

There are certain limitations associated with our study. First, as in other surveys on the subject [1–4], our questionnaire did not undergo a formal validation process before dissemination. Second, by reporting on results from the preceding round 1, peer pressure may have led to changing results in the second Delphi round to conform. Nevertheless, results from the previous round were presented in an anonymized form, thus eliminating the impact of dominant opinion leaders. Third, bias in our process of expert selection cannot be excluded and despite a very high response rate compared with other surveys, only seventy-two European experts in UGI-surgery participated, which potentially limits the relevance of our results.

Other limitations include the definition of the index procedure. Although classification of HH into four types according to Skinner and Belsey [27, 28] is broadly accepted, major uncertainties remain, particularly regarding an inconsistent and synonymous use of the terms “type II or III HH”, “mixed HH”, “large HH”, “pHH”, “upside-down stomach”, and “(intra)thoracic stomach”. Thus, in the US, the term “pHH” generally refers to all large HH (types I-IV) with migration of the fundus into the mediastinum, whereas many European surgeons strictly reserve this term for pHH type II (without any sliding component) independent of hernia size and of reducibility of the hernia sac [29–33]. Therefore, despite our effort to adequately define the index procedure of our survey, we cannot guarantee that all participants share a similar understanding of pHH.

In conclusion, consensus amongst European experts in UGI-surgery is limited to just a few basic components of surgical management for pHH. Whilst the observed therapeutic polypragmatism regarding many details of the procedure may simply manifest the clinical necessity to adapt to the clinical variability and complexity of pHH, it may also reflect a lack of standardization. Therefore, also considering the rapidly increasing prevalence of pHH in the ageing Western world, it may be a great opportunity for international surgical associations like the European Foregut Society (EFS) to promote well-designed clinical trials and guidelines.

## Supplementary Information

Below is the link to the electronic supplementary material.

Supplementary file1 (XLSX 18 KB)

Supplementary file2 (XLSX 14 KB)
